# Hybrid Materials Obtained by Immobilization of Biosynthesized Ag Nanoparticles with Antioxidant and Antimicrobial Activity

**DOI:** 10.3390/ijms25074003

**Published:** 2024-04-03

**Authors:** Gabriela Petcu, Elena Madalina Ciobanu, Gabriela Paun, Elena Neagu, Adriana Baran, Bogdan Trica, Andreea Neacsu, Irina Atkinson, Razvan Bucuresteanu, Alexandra Badaluta, Lia Mara Ditu, Viorica Parvulescu

**Affiliations:** 1 Institute of Physical Chemistry “Ilie Murgulescu”, Romanian Academy, Spl. Independentei 202, 060021 Bucharest, Romania; gpetcu@icf.ro (G.P.); adibaran@gmail.com (A.B.); neacsudanaandreea@yahoo.com (A.N.); iatkinson@icf.ro (I.A.); 2 National Institute for Research-Development of Biological Sciences, Centre of Bioanalysis, 296 Spl. Independentei, P.O. Box 17-16, 060031 Bucharest, Romania; 3 National Institute for Research & Development in Chemistry and Petrochemistry-ICECHIM, 202 Spl. Independentei, 060021 Bucharest, Romania; trica.bogdan@gmail.com; 4 Microbiology Department, Faculty of Biology, University of Bucharest, Intr. Portocalelor 1-3, 060101 Bucharest, Romaniabadaluta.valentina@s.bio.unibuc.ro (A.B.);

**Keywords:** supported AgNPs, biosynthesized colloids, hybrid materials, *Salvia officinalis* extract, TiO_2_-SBA-15, surface plasmon resonance, antioxidant activity, antimicrobial activity, visible light effect

## Abstract

Ag nanoparticles (AgNPs) were biosynthesized using sage (*Salvia officinalis* L.) extract. The obtained nanoparticles were supported on SBA-15 mesoporous silica (S), before and after immobilization of 10% TiO_2_ (Degussa-P25, STp; commercial rutile, STr; and silica synthesized from Ti butoxide, STb). The formation of AgNPs was confirmed by X-ray diffraction. The plasmon resonance effect, evidenced by UV-Vis spectra, was preserved after immobilization only for the sample supported on STb. The immobilization and dispersion properties of AgNPs on supports were evidenced by TEM microscopy, energy-dispersive X-rays, dynamic light scattering, photoluminescence and FT-IR spectroscopy. The antioxidant activity of the supported samples significantly exceeded that of the sage extract or AgNPs. Antimicrobial tests were carried out, in conditions of darkness and white light, on *Staphylococcus aureus*, *Pseudomonas aeruginosa*, *Escherichia coli* and *Candida albicans*. Higher antimicrobial activity was evident for SAg and STbAg samples. White light increased antibacterial activity in the case of *Escherichia coli (E. coli)* and *Pseudomonas aeruginosa (P. aeruginosa)*. In the first case, antibacterial activity increased for both supported and unsupported AgNPs, while in the second one, the activity increased only for SAg and STbAg samples. The proposed antibacterial mechanism shows the effect of AgNPs and Ag^+^ ions on bacteria in dark and light conditions.

## 1. Introduction

Bionanotechnology has developed as a new field, in the last decade, due to the application potential of nanosized materials with specific functions [1,2,3,4,5]. The use of plants and especially plant extracts in nanoparticle synthesis has proven to have advantages such as fast preparation and non-toxicity [2,6,7]. Thus, through a green synthesis method mediated by plants, nanoparticles with various shapes and sizes have been stabilized. The biological reduction method is one of the most extensively discussed eco-friendly techniques developed for producing silver nanoparticles. The advantages of this method are its low cost, speed, eco-friendliness, sustainability and suitability for biological applications [8,9,10,11,12,13,14,15,16]. Previous studies have shown that the size, morphology, stability and biological properties of metallic nanoparticles are strongly influenced by the types of molecules found in plant extracts and by the experimental conditions. The activity of silver nanoparticles depends on the bioavailability of silver ions and is enhanced by the presence of plant extract biocompounds. Previous researchers have shown that the shape, size and surface coating of AgNPs greatly influence their physical interactions with plant cells [17], and researchers have emphasized the importance of the surface coating used for AgNP stabilization.

The green synthesis of AgNPs using plant extracts is based on the capacity of phytochemicals present in these extracts to reduce Ag^+1^ ions to Ag^0^ and further to stabilize the new silver species by surrounding them. It has been reported that flavonoids, alkaloids, polyphenols, terpenoids, heterocyclic compounds and polysaccharides are generally responsible for obtaining metallic nanoparticles since they act as bio-reductant, capping and stabilizing agents [18]. The extant results demonstrate that in conditions of biological synthesis of green AgNPs, phytonanotechnology has the advantage of safe synthesis and applications. Furthermore, the benefits of biosynthesized silver nanoparticles for biocide purposes could be increased by using medicinal plant extracts that contain biomolecules with therapeutic activity. Furthermore, the immobilization of bioactive compounds in inorganic silica matrices can make them more effective and stable [19]. There are several studies regarding hybrid materials based on silica matrices as delivery systems for different natural biocompounds with excellent biological applications [20,21,22]. Medicinal plants provide pharmacologically active substances, and silver nanoparticles have a large surface with reactivity towards these compounds, making them effective antimicrobial agents. *Salvia officinalis* (family Lamiaceae) has exhibited a variety of medicinal effects, including antioxidant, inhibitory, anti-hyperglycemic and anti-inflammatory properties, due to its contents of bioactive compounds such as terpenoids, phenolic acids, flavonoids, tannins, volatile oils and steroids [23]. Sage extracts also have strong antibacterial and antifungal activity and are promising agents for the green synthesis of AgNPs [24,25,26]. There is great interest among researchers in the use of silver nanoparticles due to their broad antibacterial properties against many antibiotic-resistant microbial strains, high availability and low toxicity against human cells [11,27,28,29]. Although Ag is considered to have the best plasmonic characteristics among the noble metals, its stability remains a challenge due to its high surface reactivity [30]. This problem can be solved by stabilizing the surface using plant extracts. An increase was noted in the antibacterial properties of AgNPs under visible light irradiation, correlated with a higher reactive oxygen species (ROS) content due to local surface plasmon resonance stimulation [31]. In these conditions, electrons are generated that interact with substrates, giving rise to ROS. In the absence of light irradiation, the antibacterial activity of silver nanoparticles was attributed to Ag^+1^ species that could be released into the system by an oxidative dissolution process [32,33]. Thus, positively charged silver ions will interact with negatively charged components found in the structure of a microorganism, leading to metabolic damage that ultimately causes cell death [27]. Previously, plasmonic AgNPs were deposited onto Degussa (P25) titanium dioxide to improve the photocatalytic properties under visible light irradiation by lowering the activation energy [34,35]. However, there are few papers that have evaluated the plasmonic effect of AgNPs, standalone and with TiO_2_ support, on antibacterial properties [34,36]. Among these, some have shown the effect of light on antibacterial activity [37,38]. Immobilization caused an increase in photocatalytic performance and the heterogenization of photocatalytic reactions, allowing for the reuse of AgNPs/Degussa nanocomposites.

The increase in antibiotic resistance among bacteria has generated a need for new materials with antimicrobial activity [39,40]. Special interest has been directed toward inorganic materials that can be activated by various stimuli and can be combined with various other component properties, thus allowing composite materials with new and synergistic properties to be obtained. For instance, photocatalytic nanoparticles, such as those of titanium dioxide, are strongly antimicrobial upon irradiation due to the generation of highly reactive oxygen species, which, in turn, destabilize bacterial membrane components, such as phospholipids and lipopolysaccharides [41,42]. The physical interaction between nanomaterials and the cell wall membrane and their toxicity to bacteria determine the antibacterial effect. In the published studies the antibacterial activity of materials was attributed to their size, oxidation capacity, surface defects, dispersibility, composition and the synergetic effects of their components. Nanoparticles with antibacterial activity can induce spontaneous ROS generation on their surface because of their chemical and surface characteristics. Various ways for the generation of ROS by nanoparticles such as graphite, graphite oxide, graphene oxide, reduced graphene oxide, ZnO, TiO_2_, Ag-graphene oxide and zinc oxide/graphene oxide composites have been proposed [43,44,45,46,47]. Based on these observations a hypothetical mechanism for cellular toxicity could be through the generation of OH•, O_2_− and H_2_O_2_ in bacterial cells, leading to oxidation of polyunsaturated phospholipids. Also, ROS generation was correlated with DNA damage and cell death as an important pathway of oxidative stress. For graphene-based materials, it was suggested that the antibacterial mechanism is the result of a synergic effect of membrane stress and oxidative stress [43,44]. In addition, the antibacterial activity of the ZnO/GO composites was attributed to the synergistic effect of ZnO and GO. Thus, the intimate contact of *E. coli* cells and ZnO NPs on GO sheets enhanced the permeability of the bacterial membrane and the local free zinc concentration around bacteria. In the case of zinc oxide and dark conditions, the mechanism of antibacterial activity was attributed mainly to ROS originating from the interaction of water/moisture with superoxide species which are facilitated by surface defects [47].

The incorporation of TiO_2_ in mesoporous silica nanoparticles induced effects comparable with those of free titanium dioxide on supported lipid membranes, including membrane thinning, removal of lipids and formation of a partially disordered membrane outer shell [41]. The effects of ROS generated by TiO_2_ and of mesoporous silica surface morphology (smooth or spiky) on membrane destabilization were thus highlighted. Membrane destabilization was found to be minor in the absence of light and significantly increased under UV light irradiation. The antibacterial activity of TiO_2_ has been correlated in many studies with its photocatalytic properties [48,49], highlighting the effects of the dioxide nanoparticles’ size, morphology and structure. Thus, it was proven that the anatase form of TiO_2_ has the highest antimicrobial activity [48,50,51]. A high antibacterial activity, even in the absence of light, was also highlighted for the rutile form of TiO_2_ modified with other cations (Ca, Cu) [52].

Here, AgNPs were biosynthesized using sage (*Salviaofficinalis*) extract. Although there is an increased interest in the biosynthesis of silver nanoparticles [2,5,6,7,8,9,10,11,12,13,14,23,24,25,26,34], there are not enough studies regarding their immobilization on various materials and their effects on biological activity. Therefore, the current study aims to evaluate the antibacterial and antioxidant behavior of the hybrid samples obtained by immobilization of biosynthesized AgNPs on SBA-15 mesoporous silica modified with TiO_2_ 10 wt.% from different sources (impregnated titanium butoxide, commercial P25, industrial TiO_2_ rutile), compared to non-immobilized silver nanoparticles. Silica was selected as a support in order to decrease the allergic tendency of silver nanoparticles, as has been reported in the literature [53]. The properties of AgNPs and the effect of supports on them were evaluated by X-ray diffraction, UV-Vis, photoluminescence, FT-IR spectroscopy and TEM microscopy. Also, the physical properties of the synthesized materials, such as size (hydrodynamic diameter) and zeta potential, were investigated. The antioxidant activity of AgNPs was spectrophotometrically determined. The antimicrobial properties were assessed, in conditions of darkness and white light, on three standard strains: Gram-positive (*Staphylococcus aureus* ATCC 25923), Gram-negative (*Pseudomonas aeruginosa* ATCC 27853 and *Escherichia coli* ATCC 25922) and yeast (*Candida albicans* ATCC 10231) strains. The novelty of this study consists in the synthesis of new hybrid nanostructures and their antimicrobial properties evaluated comparatively in dark and light conditions.

## 2. Results and Discussion

### 2.1. X-ray Diffraction

**Figure 1:**
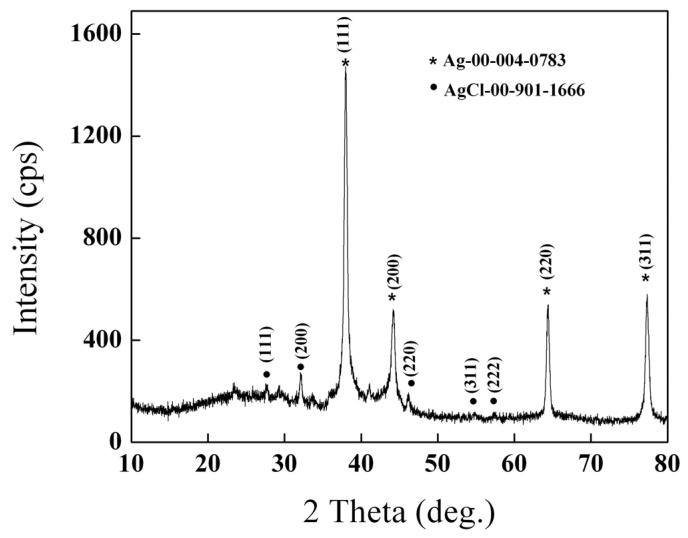
X-ray diffractogram of biosynthesized Ag NPsAgNPs.

The crystalline nature of AgNPs was evidenced by powder X-ray diffraction. Figure 1 shows high diffraction peaks at 2θ ~ 38°, 45°, 65°, and 78°, indexing the Bragg reflection planes (111), (200), (220) and (311). These results confirm the presence of a face-centered cubic structure of crystalline silver nanoparticles.

### 2.2. UV-Vis Absorption Analysis

**Figure 2:**
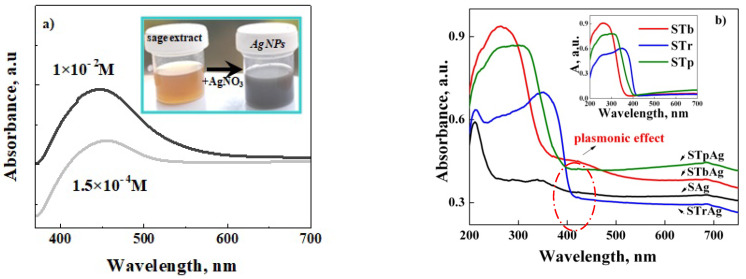
UV-Vis absorption spectra of biosynthesized AgNPs (**a**) and of supports before and after immobilization of biosynthesized AgNPs (**b**).

The formation of biosynthesized AgNPs was demonstrated by the appearance of a surface plasmon resonance (SPR) band at around 450 nm (Figure 2a). UV-Vis spectra were recorded for AgNPs obtained in conditions of different concentrations of AgNO_3_ solution (1 × 10^−2^ M and 1.5 × 10^−4^ M and 24 h of solution aging).

The primary observation of silver ion reduction and AgNPs formation was supported by the color change of the reaction mixture from light brown to gray (the inset of Figure 2a).

After the immobilization of AgNPs, a decrease and a shift to a lower wavelength were recorded for the SPR absorption band. This may be the effect of the significant decrease in AgNPs concentration and the interaction with titanium dioxide from the support. Unfortunately, the characteristic peak of the plasmonic effect is only visible for the sample obtained on the support modified with TiO_2_ by titanium butoxide solution impregnation (post-synthesis method) (Figure 2b). These results show that the interaction is stronger with titanium dioxide obtained on the silica surface (STb support) compared to P25 (STp support) or rutile (STr support). For the first support, previous studies showed, by Raman spectra, a highly dispersed state of titania and the presence of Ti–O–Si bonds, while P25 is a mixture of anatase and rutile [54]. The high values of the absorption bands recorded between 250 and 400 nm for the samples with supported TiO_2_ are assigned to titania, as can also be seen in the case of supports before AgNPs immobilization (the inset spectra of Figure 2b). A decrease in the band gap energy was obtained by immobilizing the biosynthesized silver nanoparticles, as shown in Table 1.

### 2.3. TEM/EDX Analysis

**Figure 3:**
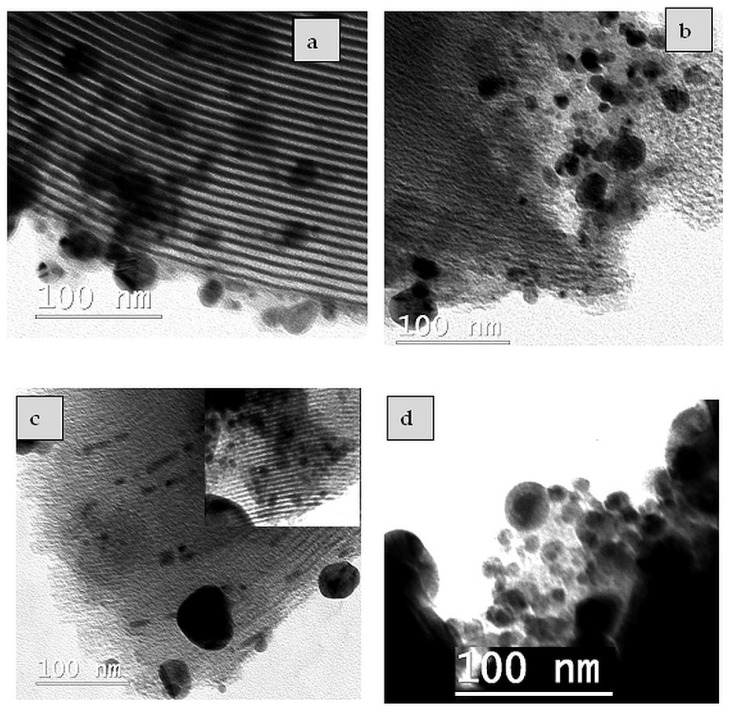
TEM images of SAg (**a**), Star (**b**), STrAg (**c**) and AgNPs (**d**) samples.

TEM analysis was used to evidence the silver nanoparticles’ morphology and dispersion on the SBA-15 mesoporous silica support modified with TiO_2_ (Figure 3). The TEM images suggested that most of the biosynthesized AgNPs have circular shapes and that only a few are elongated (Figure 3a,b). In the case of the STrAg sample, larger rutile particles can be observed, and the Ag species have elongated shapes and appear to be inside the mesoporous silica pores. In addition, the presence of spherical Ag species dispersed on the surface of the silica, similar to the other supports, can also be observed (image inserted in Figure 3c).

**Figure 4:**
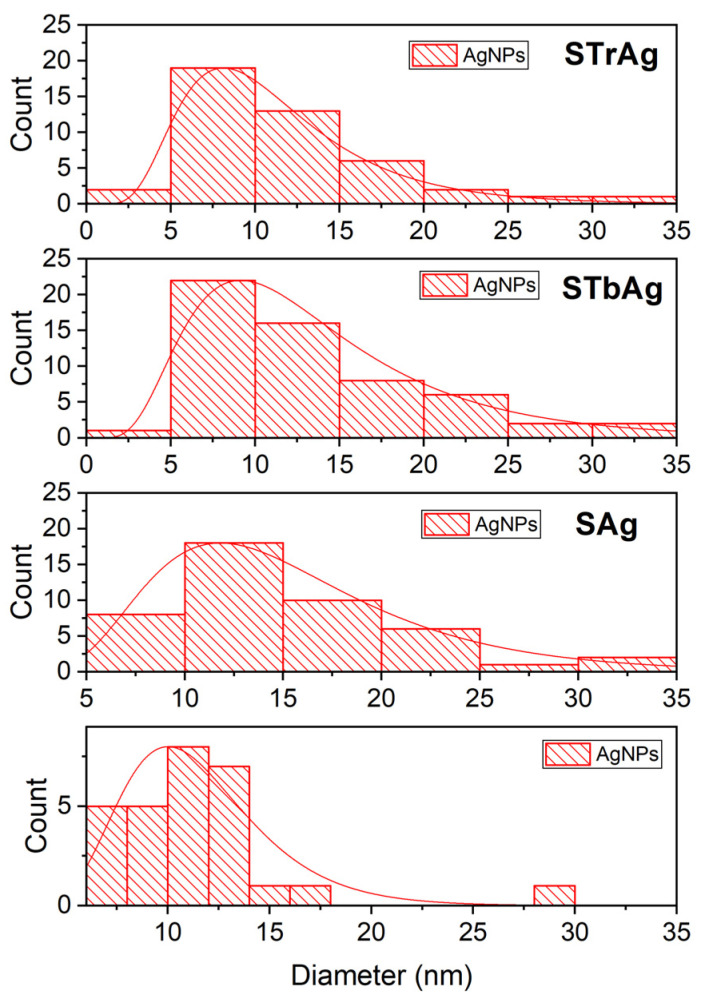
Particle size distribution curve (red line) obtained from TEM images of AgNPs before and after their immobilization.

Figure 4 shows the effect of the support on the size distribution of silver nanoparticles. It is thus observed that in the case of TiO_2_ supports, most nanoparticles have sizes between 5 and 10 nm. The number of these particles is slightly higher for the STb support obtained by immobilizing TiO_2_ by impregnating a titanium (IV) butoxide solution in ethanol. In the absence of TiO_2_, the size of the nanoparticles increases, the maximum number having dimensions between 10 and 15 nm. For unsupported AgNPs, the size distribution is narrower, so the vast majority of them have sizes up to 15 nm.

**Figure 5:**
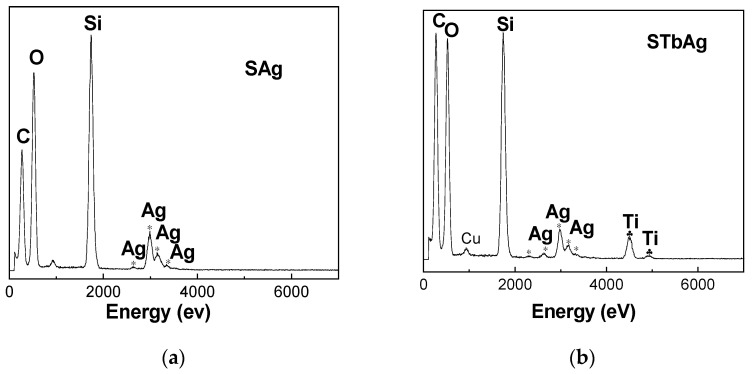

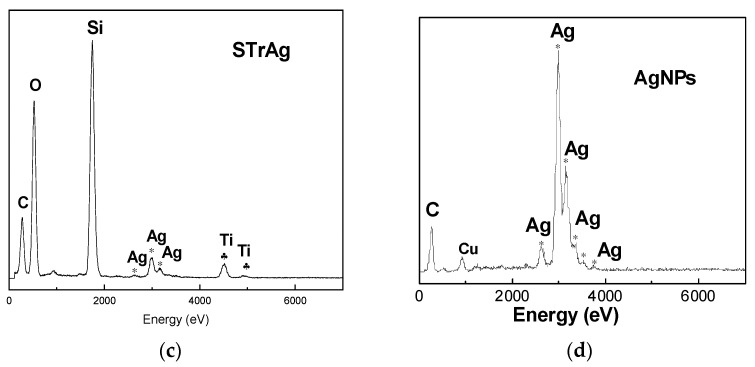
EDX images of (**a**) SAg, (**b**) STbAg, (**c**) STrAg and (**d**) AgNPs samples.

TEM image (Figure S1) indicated that TiO_2_ is not observed as a separate phase in STb sample, which is in agreement with previous studies [54,55]. The commercial TiO_2_ particles (rutile and P25 Degussa) present in the STr and STp supports were dispersed among the particles of SBA-15. These TiO_2_ materials were already characterized [52,56]. It was observed that these titanium oxides contain mixtures of particles with variable and much larger sizes (50–250 nm) with spherical (P25) or ovoid (rutile) morphology. A TEM image of the STrAg sample area (Figure S2) shows the presence of large particles next to those typical of SBA-15, which can be attributed to TiO_2_. The presence of Ti was evidenced in all SBA-15 supports modified with TiO_2_ (Figure 5 and Figure S4). EDX has also confirmed the presence of Ag species in all the samples.

### 2.4. FT-IR Analysis

**Figure 6:**
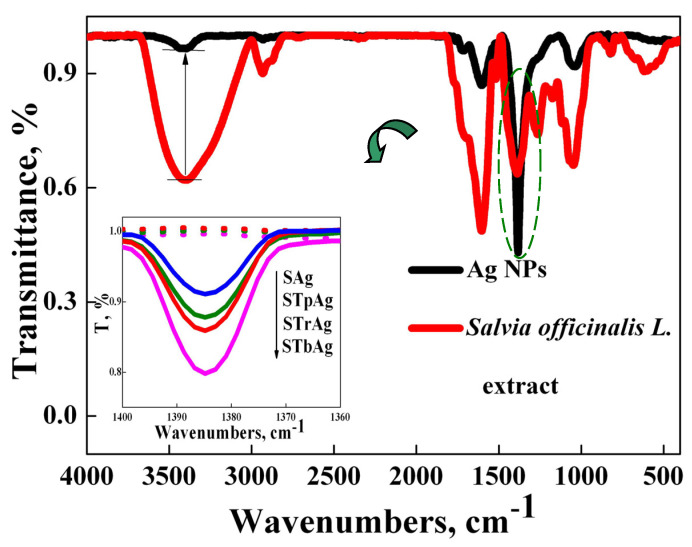
FT-IR spectra of sage extract, biosynthesized AgNPs and hybrid nanostructures (black line for AgNPs, red line for sage extract, blue line for SAg, green line for STpAg, red line from the inset image for STrAg and magenta line for STbAg; green dashed circle highlights the sharp peak recorded at 1387 cm^−1^ for AgNPs).

The biosynthesis of silver nanoparticles using an *S. officinalis* alcoholic extract was also analyzed by FTIR spectroscopy in order to follow the transformations that occur after the reduction of Ag^+^ to Ag^0^ and then the stabilization of metallic silver species by the phytochemicals present in the extract (5558.7 μg CAE/mL, as spectrophotometrically determined). FTIR spectra of the *S. officinalis* alcoholic extract and of the biosynthesized silver nanoparticles before and after immobilization on the three TiO_2_-SBA-15 supports are shown in Figure 6.

The major vibration bands in the FTIR spectrum of the *S. officinalis* extract are recorded at the following wavelengths: 3450 cm^−1^ (assigned to stretching vibration of hydroxyl group of phenols); 2932 cm^−1^, 1604 cm^−1^ and 1518 cm^−1^ (can be attributed to C=C groups in the aromatic nucleus); 1387 cm^−1^ (assigned to carbohydrates); 1263 cm^−1^ (assigned to hydroxyl groups on the aromatic nucleus) and 1048 cm^−1^ (can be attributed to the C-N group in primary amines). The bands recorded in the range 3000–2800 cm^−1^ can be attributed to the C-H and O-H groups present in carbohydrates and carboxylic acids.

Looking comparatively at the FTIR spectrum of the alcoholic sage extract and that of the biosynthesized silver nanoparticles, the preservation of the main vibration bands of the sage extract was observed, but with a lower intensity, which suggests the consumption of biomolecules during the biosynthesis of silver nanoparticles. This observation is in agreement with other reports in the literature [26] and suggests the importance of the bioactive compounds present in the used extract, acting as reducing and capping agents, in order to obtain stable silver nanoparticles. The sharp peak recorded at 1387 cm^−1^ for silver nanoparticles can be assigned to the unreacted Ag precursor (AgNO_3_) (Figure S3) and also to C-H bonding [57].

### 2.5. Photoluminescence Analysis

**Figure 7:**
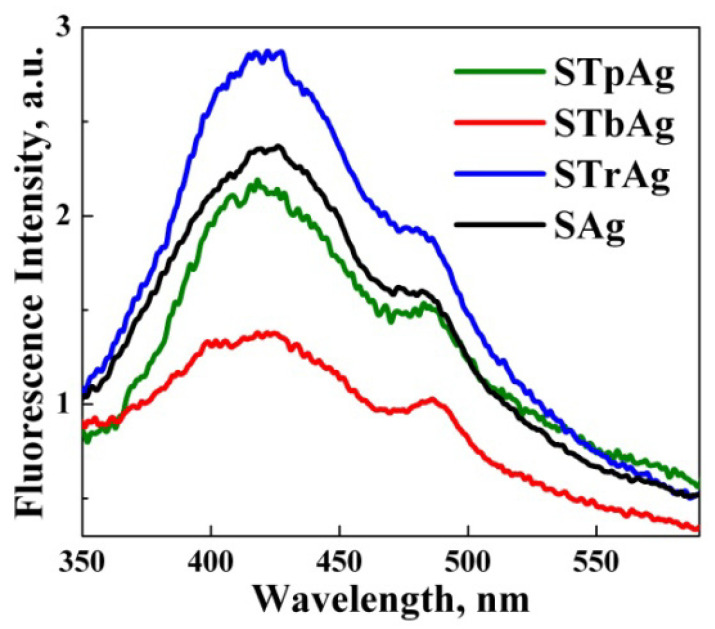
Photoluminescence spectra of hybrid nanomaterials.

The photoluminescence (PL) spectra of hybrid samples are shown in Figure 7. Excited at a wavelength of 320 nm, the samples present four major PL emission peaks at around 408, 420, 435 and 484 nm. The emission bands recorded at 408 and 435 nm are most likely due to oxygen vacancies and neutral oxygen vacancy defects’ triplet to ground transition, while the emission band recorded at 484 nm is due to surface defects [58]. The emission peaks are determined both by the phenomena that occur at the level of TiO_2_ supported on SBA-15 and by the presence of polyphenols that have a high electron density that most likely contribute to the increase in photoluminescence intensity. The slightest photoluminescent intensity recorded for the STbAg sample means the lowest recombination capacity of the photogenerated charges (e^−^/h^+^), thus suggesting the greatest number of existing defects in its structure. They were most likely created due to the synthesis method, the sample being thermally treated at 550 °C after impregnation with titanium butoxide.

### 2.6. Dynamic Light Scattering Analysis

**Figure 8:**
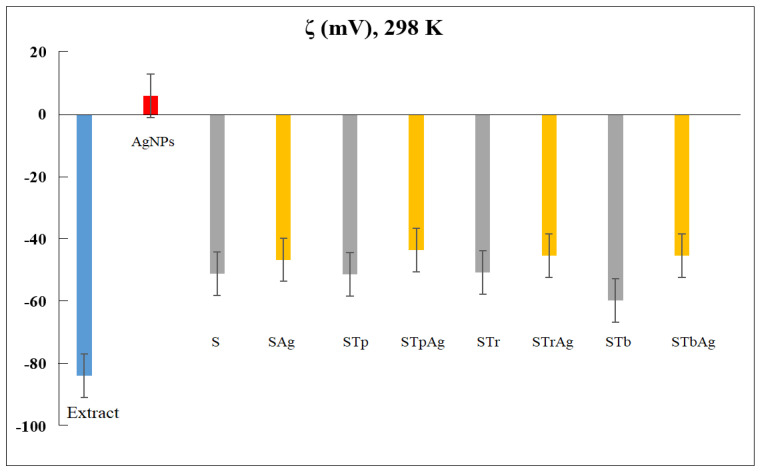

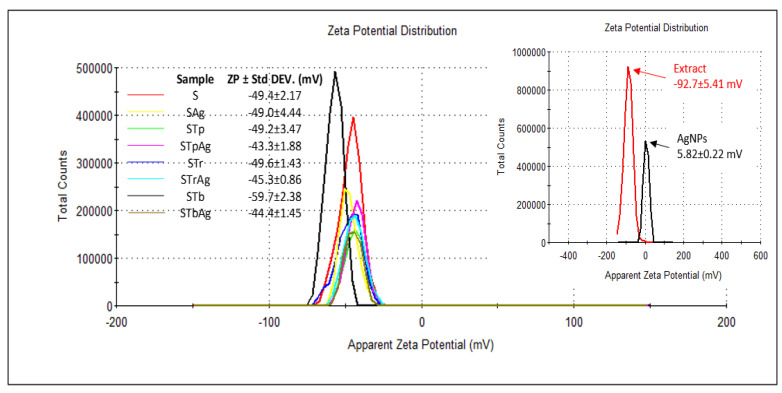
Zeta potential values and distribution curves of S, STp, STr and STb nanoemulsions in the absence and presence of AgNPs at 298 K (inset panel is for extract and AgNPs). For the extract, AgNPs and each type of support/support–AgNPs, zeta potential values are the average result for five measurements and the standard deviation.

Figure 8 comparatively presents the zeta potential (ζ) of the plant extract, AgNPs and supports (S, STp, STr, and STb) in the absence and in the presence of AgNPs.

**Figure 9:**
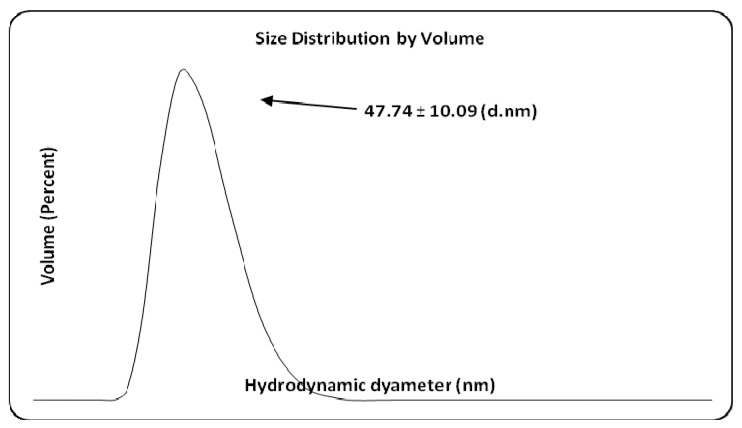
Hydrodynamic diameter distribution (in % volume) in AgNPs.

Using the same method (dynamic light scattering, DLS), the hydrodynamic diameter of AgNPs was also measured (Figure 9). The average size obtained for Ag colloidal particles is 47.74 nm. It could be observed the larger negative ζ values for the plant extract strongly increased after the reduction of Ag^+1^ ions into Ag^0^. For the supports, ζ data confirmed the poor influence of AgNPs. The variation in zeta potential is due to the modifications of the SBA-15 support with different species of TiO_2_. In addition, the STb sample was obtained by impregnation of silica with a titanium precursor (titanium butoxide) which was transformed into anatase by calcination. The impregnation of the Ti precursor solution results in a higher dispersion of it on the surface of a support compared to mechanical mixing. This explains the more negative value of the zeta potential for the STb sample. The values of the zeta potential are similar for the samples obtained by mechanical mixing. Although the STp and STr samples contain different ratios of anatase and rutile, the effect is insignificant due to the close values of the zeta potential for them. The potential value of hybrid materials is significantly influenced by the silica support. If titanium is immobilized on the support by direct synthesis, it is observed that the zeta potential becomes more negative. This could be the effect of the substitution of Si with Ti in the SBA-15 silica network [54].

Zeta potential is an indicator of nanoparticles’ stability in a suspension. Thus, the low electric charge of AgNPs explains the reduced stability of colloidal Ag particles. The stability of the obtained samples in a solution was increased by the immobilization of AgNPs on a support. The mechanism by which nanometals interact with biological systems depends on the properties of the nanometals. Among these, zeta potential is a characteristic electric charge of metal nanoparticles that governs their biological activity and their interaction with bacterial cell surfaces. Usually, the surface charge of bacteria is negative as a result of the various groups from the surface (carboxyl, phosphate, amino), the adsorption of various ions from the medium and the contribution of macromolecules from the cell wall and membrane. The interaction of bacteria with nanoparticles changes the potential of bacteria and their zeta potential. The interaction with positively charged AgNPs has the effect of changing the potential of bacteria. Although the interaction of bacteria with negatively charged surfaces is repulsive, a connection can be made through other surface mechanisms such as hydrogen bond, van der Walls, ionic and receptor–ligand interactions [53]. These interactions modify the electrical and hydrophobic properties of bacterial cells, with adverse effects on their viability. Under light irradiation, the agglomeration of AgNPs was highlighted by TEM microscopy, which led both to the modification of their zeta potential and to a decrease in antimicrobial activity [59]. These Ag nanoparticles were obtained by reduction with NaBH_4_ and stabilization with adenosine triphosphates. In the case of the biosynthesis of Ag nanoparticles, the plant extract not only achieves the reduction of silver but also prevents their agglomeration by surrounding them. Their stability and activity can be influenced by light, but the effects are specific, and immobilization on a support generates synergistic systems that can influence both their stability and their antibacterial activity.

### 2.7. Antioxidant Activity

**Figure 10:**
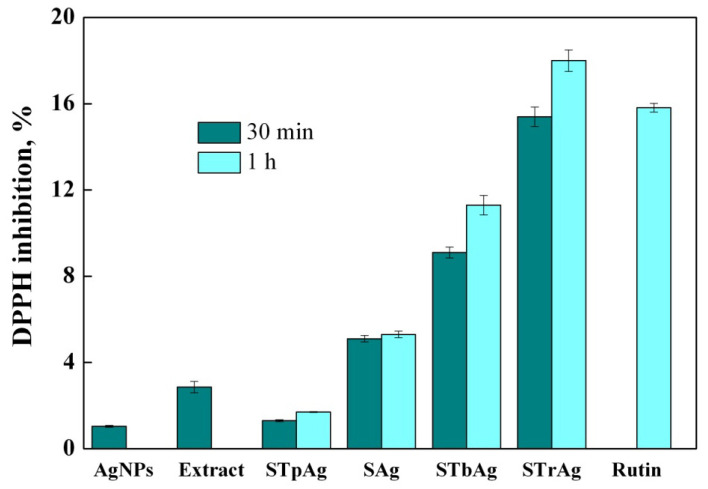
Antioxidant activity of hybrid nanomaterials.

An evaluation of antioxidant activity with the 2,2-diphenyl-1-picrylhydrazyl (DPPH) radical scavenging assay (Figure 10) proved that STrAg and STbAg had the highest scavenging activity, whereas AgNPs and STpAg showed low DPPH inhibition. The higher antioxidant activity of STb and STr supports is a result of the interaction between Ag and anatase, highly dispersed on the silica surface [54], and of rutile activity, respectively. The antioxidant activity of silica-coated TiO_2_ was previously reported [60]. The increase in antioxidant activity with time for the supported AgNPs is a result of the effect of mass transfer into the support. The increase in antioxidant activity is due to the synergistic activities of the sage extract, AgNPs and support (especially supported TiO_2_ species). The antioxidant capacity of STrAg and STbAg can be attributed to the redox potential of phenolic compounds (flavonoids and phenolic acids) from sage extract which act as reducing agents.

The high antioxidant potential of STbAg and STrAg nanocomposites can also be credited to the diverse polyphenolic compounds present in the sage extract. The interaction of polyphenolic compounds with metal ions during nanoparticle formation may result in improved antioxidant activity. Although the exact mechanism of silver nanoparticles’ antibacterial effects has not been entirely clarified, one of these mechanisms is oxidative stress which can cause damage to the cell wall of bacteria and the release of intracellular components. The antibacterial activity of silver nanoparticles is associated with ROS generation and the induction of endogenous oxidative stress in the intracellular environment [44]. Polyphenolic compounds have membrane-active properties against bacteria, which can cause leakage of cell constituents, including nucleic acids, proteins and inorganic ions such as potassium or phosphate and also act as pro-oxidants in the systems that utilize redox-active metals, such as iron and copper, thereby participating in the Fenton reaction and resulting in ROS generation [61,62].

These Ag nanoparticles synthesized with the help of plant extracts are capable of exerting antimicrobial activity through various mechanisms of action. Also, polyphenols such as flavonoids, which are known as powerful antioxidants, can suppress nucleic acid synthesis, cytoplasmic membrane function and energy metabolism [63]. On the other hand, AgNPs exhibit antimicrobial properties through multifaceted mechanisms: (1) silver nanoparticles continually release silver ions, which adhere to or pass through the cell wall and cytoplasmic membrane and lead to disruption of the cell wall and cytoplasmic membrane; (2) denaturation of ribosomes and inhibition of protein synthesis; (3) interruption of ATP production with deactivation of respiratory enzymes on the cytoplasmic membrane; (4) membrane disruption by reactive oxygen species which cause membrane disruption; (5) interference of deoxyribonucleic acid replication and prevention of cell multiplication.

### 2.8. Antimicrobial Properties

**Figure 11:**
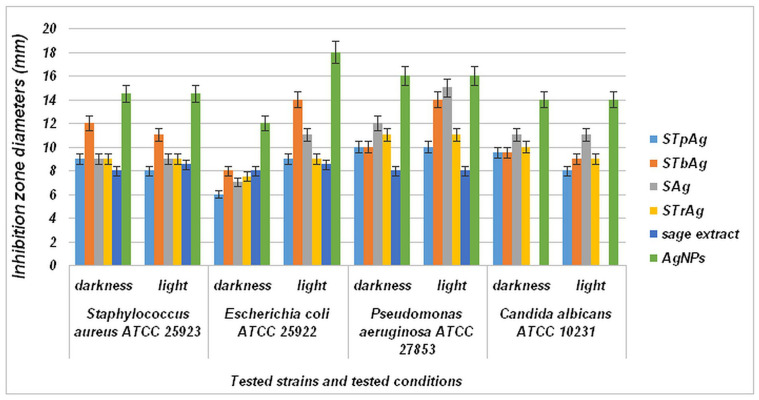
Graphic representations of the comparative results regarding the inhibition zone diameters, mm. The results are expressed as ±SD (standard deviation) and analyzed using a one-way analysis of variance (one-way ANOVA). The differences between groups were considered statistically significant when the *p*-value was <0.05.

The qualitative screening of antimicrobial effects was performed by observing the growth inhibition zone obtained after placing a sample spot on a solid medium inoculated with different microbial strains (Figures S5–S8). The results presented in Figure 11 show that although the supported samples have a significantly reduced amount of Ag compared to that of standalone AgNPs (see EDX images from Figure 5 and Figure S1b–d), they show significant antibacterial properties. The STbAg and SAg samples induced visible inhibition of microbial growth toward all tested strains, with the best results on Gram-negative strains *E. coli* ATCC 25922 and *P. aeruginosa* ATCC 27853. For these two samples, incubation in light conditions induced a more intense inhibitory effect, with the diameter of the inhibition zone increased by 25% (average) compared to the diameter obtained in dark incubation conditions (Figure 11).

Results for the antibacterial effect of the supports (STp, STb, Str, S) and solvent (ethanol 50%) indicated zero inhibition zone. At the same time, we specify that in the case of samples with supported silver nanoparticles, for which the inhibitory effect was evaluated, the amount of AgNPs was 20 times lower than that in the unsupported sample. Thus, the results presented in Figure 11 indicate a much higher activity of the supported samples. This behavior is due to the synergistic effect given by the biosynthesized silver species and the charged carriers resulting from the photo-activation of the support used for AgNPs immobilization (TiO_2_ supported on mesoporous silica SBA-15). The high number of available photogenerated species recorded for the STbAg sample, as suggested by photoluminescence analysis (Figure 7), led to a most pronounced inhibitory effect on microbial growth of the hybrid nanostructure tested under irradiation.

The effect of light on the inhibition zone diameter is more significant in the case of Gram-negative (*Pseudomonas aeruginosa* and *Escherichia coli*) bacteria. In the case of *Escherichia coli*, an increase in antibacterial activity under light can be observed for all samples. More significant is the increase for the AgNPs sample and samples of AgNPs immobilized on SBA-15 without and with TiO_2_ dispersed on silica by impregnation of an alcoholic TBOT solution. Unlike the other TiO_2_ samples (STrAg, STpAg), the STbAg sample has titanium oxide in the form of well-dispersed nanoparticles on the SBA-15 surface [64]. In the case of the other samples, the SBA-15 and oxide particles were dispersed by mixing them in the solution. Therefore, the difference between the three supports with TiO_2_ is given not only by the crystalline structure of TiO_2_ but also by the particle sizes and their dispersion. In the case of Pseudomonas aeruginosa, the significant effect of light can be observed only in the case of samples SAg and STbAg. In conclusion, the TiO_2_ immobilization method determines the antibacterial properties of supported AgNPs. Also, the advanced dispersion on the surface of TiO_2_ significantly influences the properties of Ag nanoparticles such as the antibacterial activity and the plasmonic effect evident only for the AgNPs and STbAg samples.

Besides this, the activation of the plasmonic effect of AgNPs occurs under irradiation, as evidenced by UV-Vis spectroscopy (Figure 2b) only for the STbAg sample. The stimulation of the local surface plasmon resonance (LSPR) effect of AgNPs under visible light irradiation leads to an increase in antimicrobial activity, as reported in the literature [31]. This behavior was noticed only in the case of the simultaneous presence of TiO_2_ and biosynthesized AgNPs, while for the supports containing TiO_2_-modified SBA-15 mesoporous silica (STb, STr, STp), no inhibitory effect was observed, even under light irradiation.

The presence in the synthesized nanohybrid systems of Ag^+^ ions (from AgNO_3_ precursor) that can bind via the polyphenolic compounds of the sage extract to the surface of the silver nanoparticles formed during biosynthesis cannot be excluded, as reported in the case of using sodium citrate as reducing agent [33]. Thus, the possible release of Ag^+^ ions bound to the AgNPs surface could be associated with the inhibitory effect of all synthesized materials, evidenced even in dark conditions (Figure 10, Table 2). Silver ion release was investigated for a Ag/TiO_2_/Ag/a-TiO_2_ nanocomposite film [65]. The results evidenced a long saturation time for silver ions due to their protection by a very thin titania layer. Therefore, the protection of Ag nanoparticles and their immobilization is a solution for increasing the durability of the obtained materials. Here, AgNPs are covered with a stabilizing layer of sage extract components and immobilized on a support. Obviously, the presence of bioactive compounds from sage extract that act as reducing and capping agents also makes a real contribution to the inhibitory effect of hybrid nanostructures toward all tested strains in darkness, clearly proving the antibacterial effect of the sage extract itself (Figure 11). Also, various other properties of inorganic nanoparticles such as size distribution, morphology and surface charge influence the biological activity of the samples [53,66,67].

The study of Radzikowska-Büchner (2023) showed that the antimicrobial activity of synthesized AgNPs is mediated by a nanoparticle core of appropriate size and shape and the surface modification by extract components, the strongest activity of synthesized AgNPs being observed against yeast strains [68]. In their study hypothesis, the negative charge of AgNPs does not seem to be favorable from the point of view of the contact-killing mechanism.

The relationship between ZP and the physiological state of bacteria was used to characterize their structural damage as a result of different environmental factors such as the zeta potential of a surface. Thus, the results presented in Figure 11 agree with the zeta potential values (Figure 8). It is observed that the antibacterial effect of AgNPs is almost constant in the light and in the dark for all bacteria except *Escherichia coli*, which is Gram-negative. This is more strongly influenced by the weak positive potential of AgNPs. The effect of light on the activity is significant in this case. In the case of *Staphylococcus aureus*, which is Gram-positive, the antibacterial activity is higher in the dark for the STbAg and STpAg samples, the first having the highest negative potential and both containing anatase. In the presence of light, the activity decreases slightly. These results support the effect of the interaction of bacteria with the surface of the solid with antibacterial activity in the dark.

**Figure 12:**
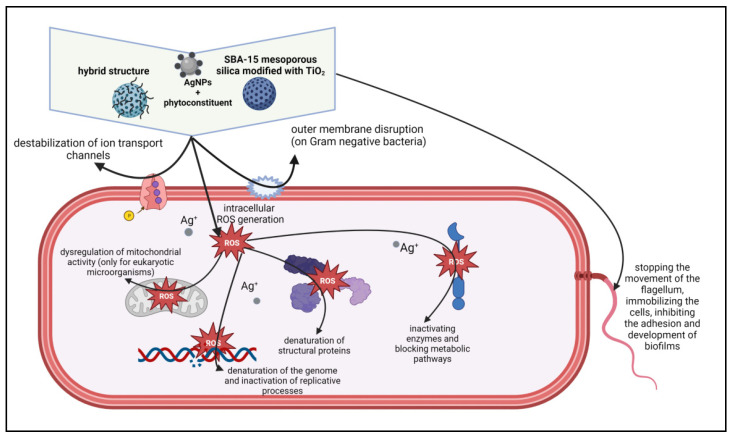
The proposed antimicrobial mechanisms of the synthesized samples (created with BioRender.com, accessed on 15 May 2023).

A significant inhibitory activity can be observed in the case of the STrAg sample against *Pseudomonas aeruginosa*. However, the activity is not influenced under light conditions. An increase in activity in the light was observed only for *Escherichia coli*. Figure 12 shows the proposed mechanism for the antibacterial activity of both colloidal Ag particles (here called AgNPs) and Ag ions.

According to existing theories [69,70,71], Ag ions can destabilize the cell membrane, even creating holes through which they penetrate into the cell. They interact with the thiol (-SH) groups of proteins and enzymes. At the same time, AgNPs can adhere to the cell membrane and generate voids through which they penetrate into the cell and then interact with its components. These processes take place in the dark, which explains the inactivation of the bacteria. The proposed mechanism is in accord with previous studies [72] which suggested two effects of AgNPs’ action: inhibition of cell multiplication by membrane damage and cell destruction by ROS effects. In addition, TiO_2_ nanoparticles can increase peroxidation of the lipid membrane, disrupting cell respiration.

The quantitative results expressed by minimal inhibitory concentration (MIC) values allowed the quantitative evaluation of the tested compound, in the same two inhibitory conditions. The MIC values of the supported samples presented in Table 2 were reported to the amount of AgNPs from each sample. The differences between the obtained MIC values obtained in the two incubatory conditions (dark and light) confirmed the qualitative results, with lower values in light conditions for the samples STbAg and SAg (Table 2).

An effect of light on MIC values can also be observed for the STpAg sample, but it is selective for *Staphylococcus aureus* and *Candida albicans*. The results presented in Table 2 evidence a decrease in MCI values for immobilized samples for almost the majority of the experiments. The antibacterial properties of the obtained hybrid nanomaterials are the result of a synergistic effect of the Ag and TiO_2_ species, the sage extract and the mesostructured silica support.

Compared to the MIC values obtained for the standard antibiotics (according to CLSI 2023), most of the MIC values of the tested samples were 3–4 times higher, especially for bacterial strains. Instead, samples SAg and STrAg demonstrated a very good efficiency against the yeast strain *C. albicans* ATCC 10231 (MIC = 1.71 µg/mL), compared to fluconazole (MIC = 4 µg/mL), in both incubation conditions. Fluconazole is a fungistatic antibiotic and one of the most commonly prescribed antifungal drugs for *Candida* infections; it acts as an inhibitor of the cytochrome P450 enzyme lanosterol demethylase (14α-demethylase), generating cell membrane damage [73]. As a consequence of its fervent clinical recommendation, many *C. albicans* isolates have gained resistance to fluconazole, based on different molecular mechanisms (drug target gene *erg11* alteration, efflux pump overexpression) [74]. By comparing the fluconazole mechanism of action and the inhibitory effect expressed by SAg and STrAg, we can also explain the antifungal mechanism of Ag ions through destabilization of the yeast cell wall structure and permeability, including surface shrinkage, cell aggregation, pit and pore formation, and general deformation [75].

The results of the control tests of the antibacterial activity of the Ag nanoparticles isolated from the supernatant (AgNPs i) indicated (Table 3, Figure S9) a small decrease ofcrease in the inhibition zone diameters compared to the initially obtained Ag nanoparticles (AgNPs).

This indicates the stability of the AgNPs due to the strong interaction between Ag and the templates from the sage extract.

## 3. Materials and Methods

### 3.1. Material Preparation

#### 3.1.1. Chemicals

Tetraethyl orthosilicate (TEOS), Pluronic P123 (PEO20PPO70PEO20, average molecular weight 5800) and hydrochloric acid (HCl) used for the synthesis of SBA-15 were purchased from Sigma-Aldrich (St. Louis, MO, USA). Silica was modified using titanium (IV) butoxide, n-propanol from Sigma-Aldrich, Tytanopol TiO_2_ microparticles and commercial P25. Silver nitrate (AgNO_3_) used for the biosynthesis of AgNPs, ethanol (96.3% *v*/*v*) used for obtaining the sage extract, Folin–Ciocalteu reagent, aluminum chloride (AlCl_3_) and 2,2-diphenyl-1-picrylhydrazyl (DPPH•) were also obtained from Sigma-Aldrich.

The medicinal plant was acquired from a national producer (SC STEF MAR SRL, Valcea, Romania) of herbal teas in dry form. The plants were collected from Valcea County (Romania).

#### 3.1.2. Synthesis of TiO_2_-Modified SBA-15 Supports

Siliceous SBA-15 was synthesized as we reported before [54], using an amphiphilic triblock copolymer (Pluronic P123), HCl aqueous solution and TEOS as a silica precursor. SBA-15 mesoporous silica was further modified with TiO_2_ 10 wt.% from different sources (titanium butoxide, commercial TiO_2_ P25 and industrial TiO_2_ rutile).

SBA-15 mesoporous silica was impregnated with TiO_2_ from an alcoholic solution of titanium (IV) n-butoxide by the incipient wetness impregnation method. After impregnation, the resulting material was kept for 24 h at room temperature, dried at 80 °C and calcinated in air at 550 °C. The sample thus obtained was named STb. Modification of SBA-15 with commercial P25 and industrial TiO_2_ rutile was performed by homogeneously mixing the 2 powders in an aqueous medium. After 1 h, the resulting mixtures were kept for 24 h at room temperature and then dried at 40 °C. The powders thus obtained were named STp and STr.

STb, STp and STr were further used as supports for the immobilization of biosynthesized AgNPs, described below.

#### 3.1.3. Preparation of Concentrated Extract by Nanofiltration

The ethanol extract was obtained as follows: 10 g of the dry plant (flowers, leaves, and stems) finely ground was mixed with 100 mL of 50% (*v*/*v*) ethanol (EtOH) and then introduced into a sonication bath at a frequency of 35 kHz and 25 °C for 90 min. After filtration through Whatman filter paper, the extracts were processed by microfiltration (MF) through a 0.45 µm pore size membrane (Merck Millipore, Darmstadt, Germany) and nanofiltration (NF) through a 1000 MWCO membrane (Merck Millipore, Darmstadt, Germany). Membrane filtration experiments were performed using a lab cross-flow membrane filtration unit (KMS Laboratory Cell CF-1, Koch Membranen GmbH, Rimsting, Germany).

#### 3.1.4. Biosynthesis and Immobilization of AgNPs

In a typical experiment, the obtained extract was added under stirring to a 0.025 g/mL aqueous solution (1.5 × 10^−4^ M) of silver nitrate (AgNO_3_) in a 1:1 volumetric ratio. The obtained mixture was left under stirring for 24 h at room temperature. The silver nanoparticles thus prepared were tested as such (sample named AgNPs) but also immobilized by incipient wetness impregnation method (1 wt.%) on the TiO_2_-modified SBA-15 supports. The hybrid samples were named STbAg, STpAg and STrAg. In order to compare the effect of TiO_2_ on the properties of the obtained hybrid nanostructures, only SBA-15 silica was used as a support for AgNPs immobilization. This sample was named SAg. The composition of each sample is summarized in Table 4. For the control test, AgNPs without organic templates were obtained by separation using a centrifuge (10,000 rpm, 10 min). The obtained solid was dispersed in distilled water and again separated by centrifugation (sample named AgNPs i).

### 3.2. Characterization Methods

#### 3.2.1. Characterization of *Salvia officinalis* Extract

UV-Vis diffuse reflectance spectra of sage extract were recorded on a Jasco V-570 spectrophotometer (Jasco, Tokyo, Japan).

The phenolic content was spectrophotometrically determined using a Folin–Ciocalteu assay [76] and expressed as chlorogenic acid equivalents (CAE) mg/mL.

The flavonoid content was calculated using the AlCl_3_ colorimetric method as described by Lin [77] and was expressed as quercetin equivalents (QE) μg/mL.

The antioxidant activity was measured with a UV-Vis spectrophotometer (Jasco V-630), using the DPPH radical scavenging activity [78]. The decrease in the DPPH radical absorption by the antioxidant action is used to evaluate the antioxidative activity. The antiradical activity was calculated using the following equation:

% Inhibition of DPPH activity = [(A_0_ − A_s_)/A_0_] × 100

where A_0_ = blank absorbance and A_s_ = sample absorbance.

#### 3.2.2. Characterization of AgNPs before and after Immobilization

The X-ray diffraction (XRD) pattern of AgNPs was recorded using a Rigaku Ultima IV diffractometer, Tokyo, Japan, with Cu Kα (λ = 0.15406 nm) with a scanning speed of 1°/min. Phase identification was performed using Rigaku PDXL (version no 1.8, Rigaku, Tokyo, Japan) with a Whole Powder Pattern Fitting (WPPF) module, connected to the ICDD-PDF-2 database. UV-Vis diffuse reflectance spectra were recorded on the same JASCO V570 spectrophotometer. The band gap energy of samples was obtained using the Kubelka–Munk function by plotting [F(R)·hυ]^1/2^ versus photon energy (eV). The fluorescence spectra of powders were recorded with an FLSP 920 spectrofluorimeter (Edinburgh Instruments, Livingston, UK). The excitation source was a Xe lamp, the excitation wavelength was 320 nm and spectra were recorded between 350 and 600 nm. The excitation and emission slits were 10 nm for all measurements. TEM images were obtained using a TECNAI 10 G2-F30 and F20 G2 TWIN Cryo-TEM–FEI microscope with EDX (FEI, Eindhoven, The Netherlands). The hydrodynamic diameter (Z-average) and polydispersity index (PdI) of AgNPs and the zeta potential (ζ) of the supports and support–Ag formulation nanoparticles were studied by dynamic light scattering (DLS) using a Malvern ZetaSizer Nano-ZS instrument (Malvern Instruments, Malvern, United Kingdom). The nanodispersions were equilibrated prior to the measurements; the measurements were performed with 10 runs at 298 K, and the results are reported as ζ ± S.D. The measurements for AgNPs were performed considering the properties of ethanol used in solutions preparations, standard viscosity of 1.04 cP, refractive index of 1.36 and dielectric constant of 24.3. For supports and support–Ag formulations, the dispersant chosen for measurements was water, which at 298 K has a fixed viscosity of 0.8872 cP, a refraction index of 1.330 and a dielectric constant of 78.5. Fourier transform infrared (FT-IR) spectroscopy was used to identify the characteristic functional groups in the samples. A Tensor 27 Brucker FT-IR spectrometer (Bruker Co., Karlsruhe, Germany) was used to record IR spectra. A potassium bromide micro-disk was prepared from finely ground powder of a 1 mg sample with 100 mg of KBr. The scanning wavelength of infrared was at 4000–400 cm^−1^ at a resolution of 4. For antioxidant activity evaluation of hybrid nanostructures, the powders were immersed in methanol (10 mg/mL), vortexed for 15 min, and then filtered and analyzed for DPPH inhibition. The method is based on the discoloration of the stable radical DPPH (2,2-diphenyl-1-picrylhydrazyl), purple colored. In a typical experiment, 100 µL of filtrate is mixed with methanol and DPPH solution and spectrophotometrically analyzed (λ = 517 nm) after 30 min and 1 h. Rutin was used as the reference, while the methanolic solution of DPPH without nanoparticles was used for the negative control.

#### 3.2.3. Characterization of Antimicrobial Test

The antimicrobial assay was performed using standard strains, from the collection of the University of Bucharest, Microbiology Department: *Staphylococcus aureus* ATCC 25923 (Gram-positive), *Pseudomonas aeruginosa* ATCC 27853 and *Escherichia coli* ATCC 25922 (Gram-negative), and *Candida albicans* ATCC 10231 (yeast strain). For the qualitative screening of the antimicrobial activity of tested compounds, an adapted spot diffusion method was followed (according to CLSI 2022 standard) in order to determine the diameter of the inhibition zone. An amount of 20 µL solution of each compound of 30 mg/mL concentration was double-spotted on the surface of the agar medium seeded with microbial inocula. The diameter of the inhibition zone was measured and expressed as the average of the two values obtained/sample, expressed in mm.

Also, for establishing the MIC (minimum inhibitory concentration) values for the tested compounds, a binary microdilution method performed in 96-well plates was utilized. The sterile nutrient broth and Sabouraud broth medium were added into sterile 96-well plates, and binary dilutions of each tested sample were performed in a final volume of 150 μL, followed by the addition of 15 μL microbial suspension adjusted to an optical density 1.5 × 10^8^ CFU/mL to each well. Gentamicin and fluconazole were used as standard antibiotics for comparative controls, considering CMI values in accordance with the CLSI 2022 standard. The MIC values were established after 24 h of incubation at 37 °C, in two conditions (darkness and light), by visual analysis and spectrophotometric measurement (absorbance reading at 620 nm), using a BIOTEK SYNERGY-HTX ELISA multi-mode reader (BioTek, Winooski, VT, USA). Each experiment was performed in triplicate and repeated on at least three separate occasions. The schematic figure illustrating the proposed antimicrobial mechanisms of the samples was made using BioRender: Scientific Image and Illustation Software.

#### 3.2.4. Statistical Analysis

The antimicrobial activity values were statistically analyzed with GraphPad Prism 10.1 from GraphPad Software (San Diego, CA, USA). The results were expressed as ±SD (standard deviation) and analyzed using a one-way analysis of variance (one-way ANOVA). The differences between groups were considered statistically significant when the *p*-value was <0.05.

## 4. Conclusions

AgNPs were biosynthesized using sage (*Salvia officinalis*) extract, and new hybrid materials with antioxidant activity and antimicrobial properties against a broad spectrum of microorganisms (Gram-positive, Gram-negative and yeast strains) were obtained. The surface plasmon resonance effect specific to metallic silver nanoparticles was evidenced after the immobilization of biosynthesized AgNPs on silica modified with TiO_2_ using titanium butoxide as a precursor. This optical property led to obtaining the most pronounced inhibitory effect on microbial growth under irradiation. A higher antibacterial property under irradiation was evidenced for all the synthesized hybrid nanostructures and is a result of the synergistic effect of photo-activated AgNPs and TiO_2_, biocomponents from the sage extracts with antibacterial properties, and last but not least, the use of mesoporous silica to support all three active components. In these conditions, reactive oxygen species are generated and induce high oxidative stress on cells which finally leads to microbial death. A synergistic effect of AgNPs and the support was highlighted in the case of both antioxidant and antibacterial properties. The inactivation of bacteria in dark conditions was explained by the effect of zeta potential on their interaction with the surface of particles containing AgNPs. Thus, the interaction of a Gram-positive bacterium such as *Staphylococcus aureus* with the surface of a sample with a strongly negative value of zeta potential resulted in a higher activity in the dark than in the light. This can be explained by the effect of zeta potential which may have a slightly greater influence on Gram-positive bacteria activity than the reactive oxygen species formed in the light. These results suggest future directions such as the effect of light on the materials’ zeta potential as well as the stability of their antibacterial effect.

## Figures and Tables

**Table 1:** The optical band gap energy value of samples.

Sample	Eg (eV)
STp	3.11
STpAg	3.08
STr	2.95
STrAg	2.91
STb	3.45
STbAg	3.20

**Table 2:** Quantitative results of antimicrobial activity (minimal inhibitory concentration, µg/mL) (STD Dev = 0) (the bolded words in the first column are the samples tested).

	*Staphylococcus* *aureus* ATCC 25923		*Escherichia* *coli* ATCC 25922		*Pseudomonas* *aeruginosa* ATCC 27853		*Candida* *albicans* ATCC 10231	
	Darkness	Light	Darkness	Light	Darkness	Light	Darkness	Light
**STpAg**	54.99	27.49	27.49	27.49	13.74	13.74	3.43	1.71
**STbAg**	6.87	6.87	13.74	6.87	13.74	6.87	13.74	0.84
**SAg**	27.49	27.49	13.74	13.74	27.49	13.74	1.71	1.71
**STrAg**	13.74	27.49	13.74	13.74	13.74	13.74	1.71	1.71
**Sage extract**	139	139	2780	139	556	556	278	278
**AgNPs**	47	23	23.4	23.4	23.4	23.4	23.4	23.4
**Gentamicin**	2	2	4	4	4	4	-	-
**Fluconazole**	-	-	-	-	-	-	4	4

**Table 3:** Inhibition zone diameters (mm) (expressed as average of the three values obtained for each fraction).

Bacterial Strain	AgNPs	AgNPs i
*E. coli* ATCC 25922	12	10.6
*S. aureus* ATCC 25923	14.5	12

**Table 4:** Summary information about synthesized samples.

Sample	Support	TiO_2_ Phase	AgNPs
STp	SBA-15	Anatase + Rutile 10 wt.%	-
STb	SBA-15	Anatase 10 wt.%	-
STr	SBA-15	Rutile 10 wt.%	-
SAg	SBA-15	-	Biosynthesized AgNPs 1 wt.%
STpAg	SBA-15	Anatase + Rutile 10 wt.%	Biosynthesized AgNPs 1 wt.%
STbAg	SBA-15	Anatase 10 wt.%	Biosynthesized AgNPs 1 wt.%
STrAg	SBA-15	Rutile 10 wt.%	Biosynthesized AgNPs 1 wt.%

## Data Availability

The data presented in this study are available on request from the corresponding author.

## Supplementary Materials

The following supporting information can be downloaded at https://www.mdpi.com/article/10.3390/ijms25074003/s1.
