# Supplementary material for: Hybrid Materials Obtained by Immobilization of Biosynthesized Ag Nanoparticles with Antioxidant and Antimicrobial Activity
Source: Int J Mol Sci. 2024 Apr 3;25(7):4003. doi: 10.3390/ijms25074003 (PMC11012143; doi:10.3390/ijms25074003)
Supplement: Supplementary file 1 [file ijms-25-04003-s001.zip › ijms-2867849-supplementary.pdf]

International Journal of Molecular Sciences

Supplementary Materials

Hybrid materials obtained by immobilization of biosynthesized Ag nanoparticles with antioxidant and antimicrobial activity

Gabriela Petcu [1], Madalina Ciobanu [1], Gabriela Paun [2], Elena Neagu [2], Adriana Baran [1], Bogdan Trica [3], Andreea Neacsu[1], Irina Atkinson[1], Razvan Bucuresteanu [4], Alexandra Badaluta[4], Lia Mara Ditu [4] and Viorica Parvulescu [1,*]

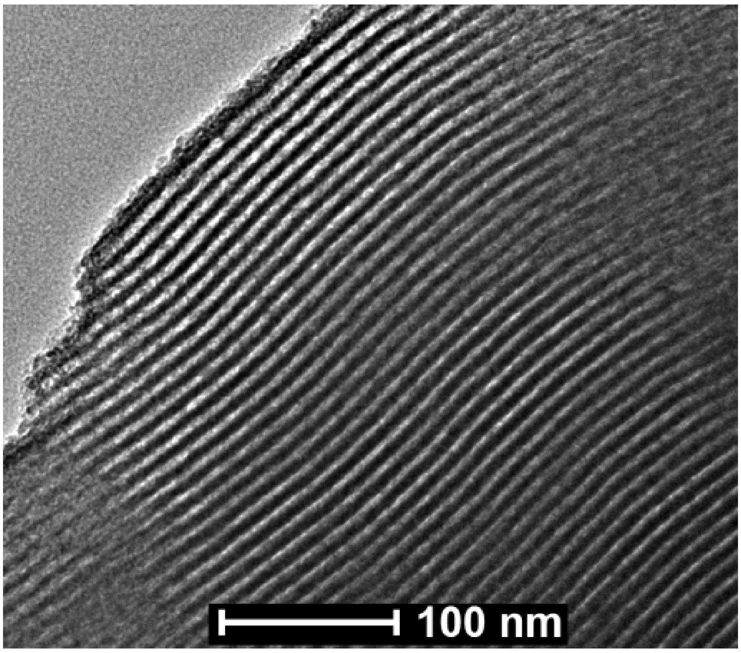

Figure S1. TEM image of STb support

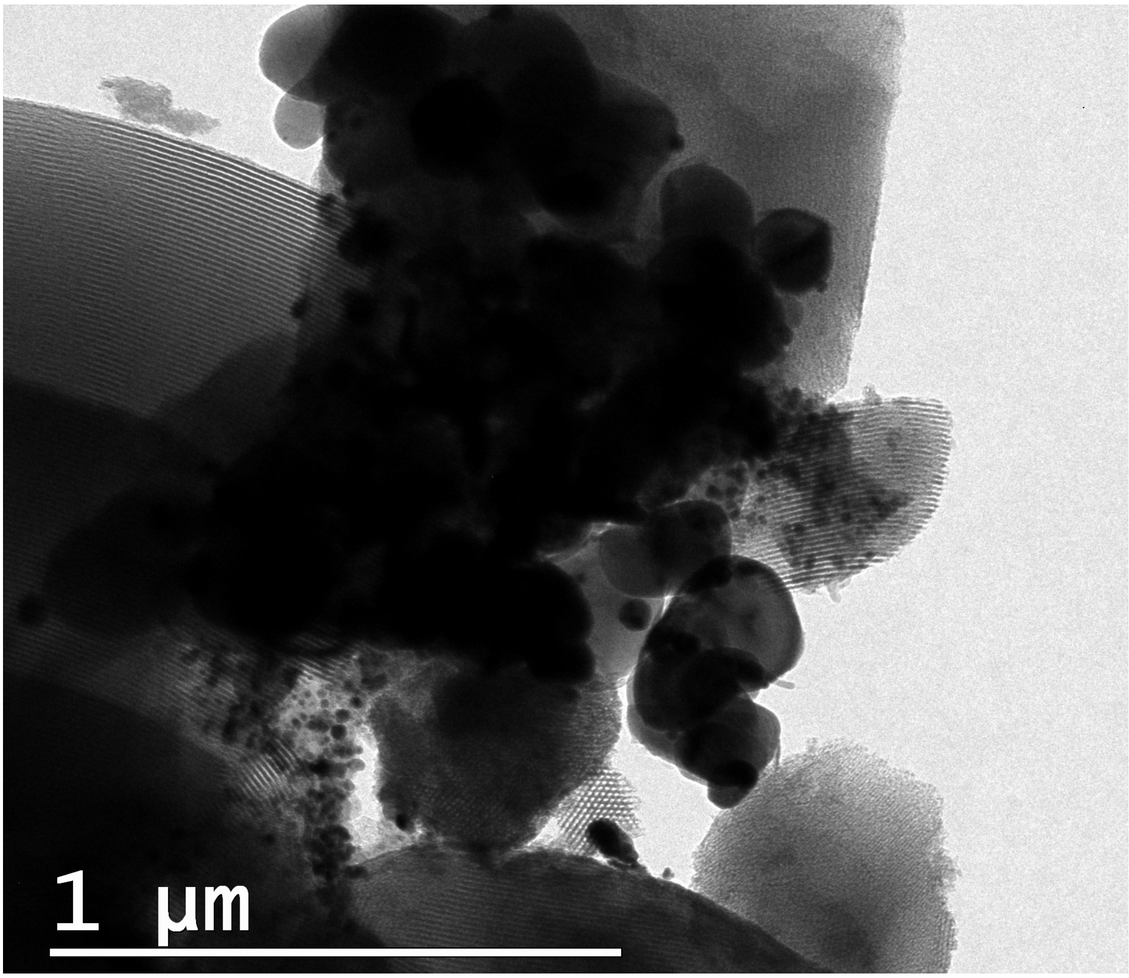

Figure S2. TEM image of STrAg sample

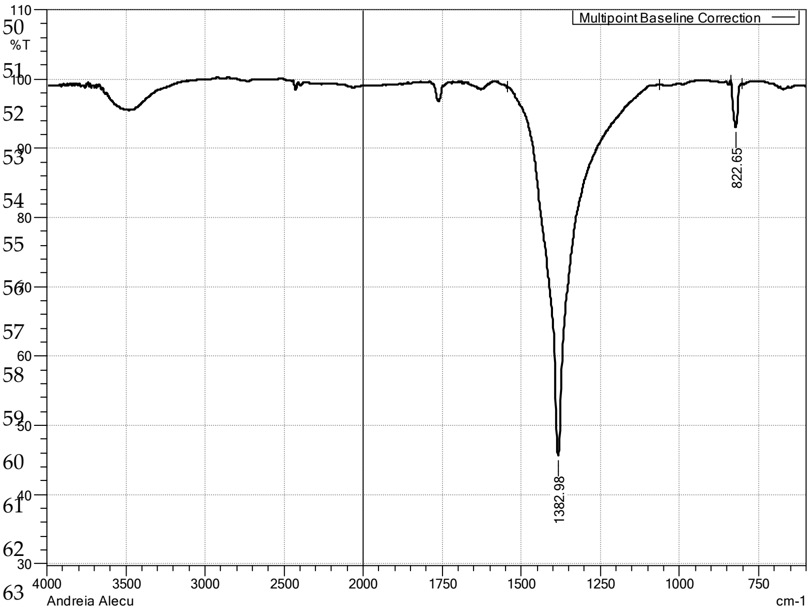

Figure S3. FT-IR spectrum of $AgNO_3$.

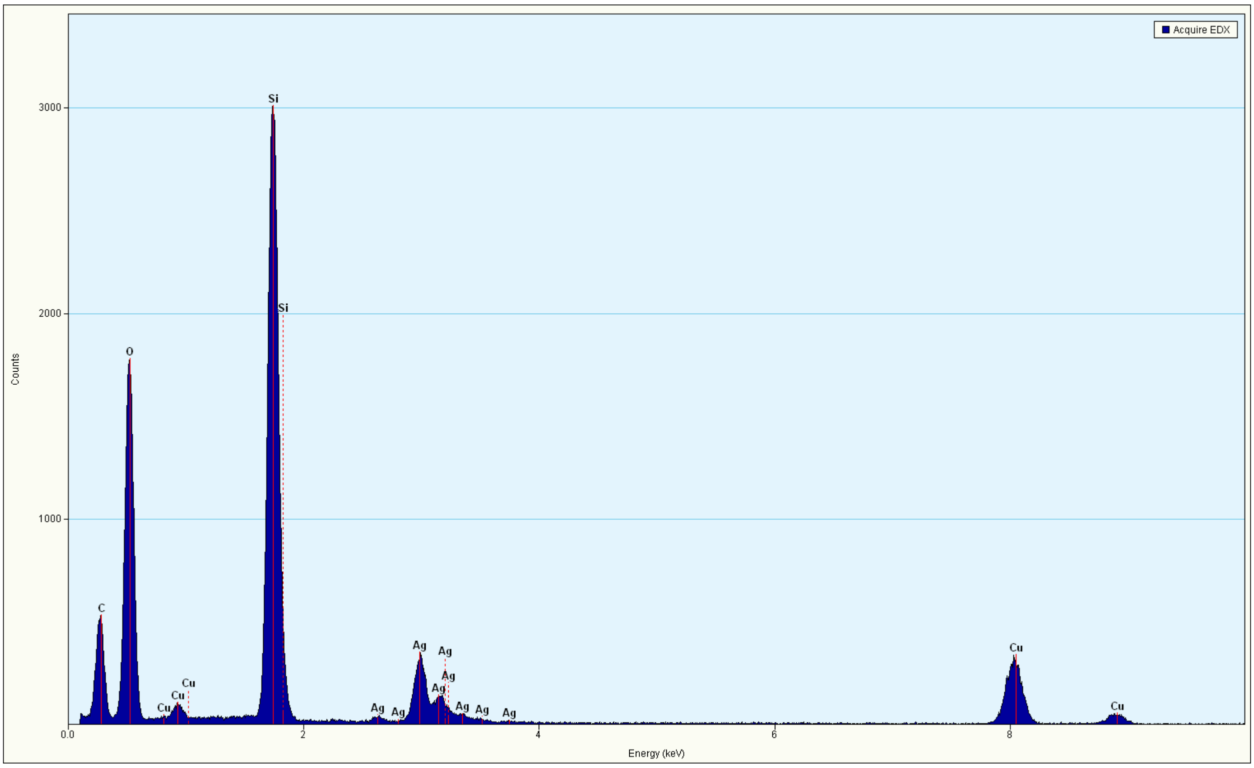

a)

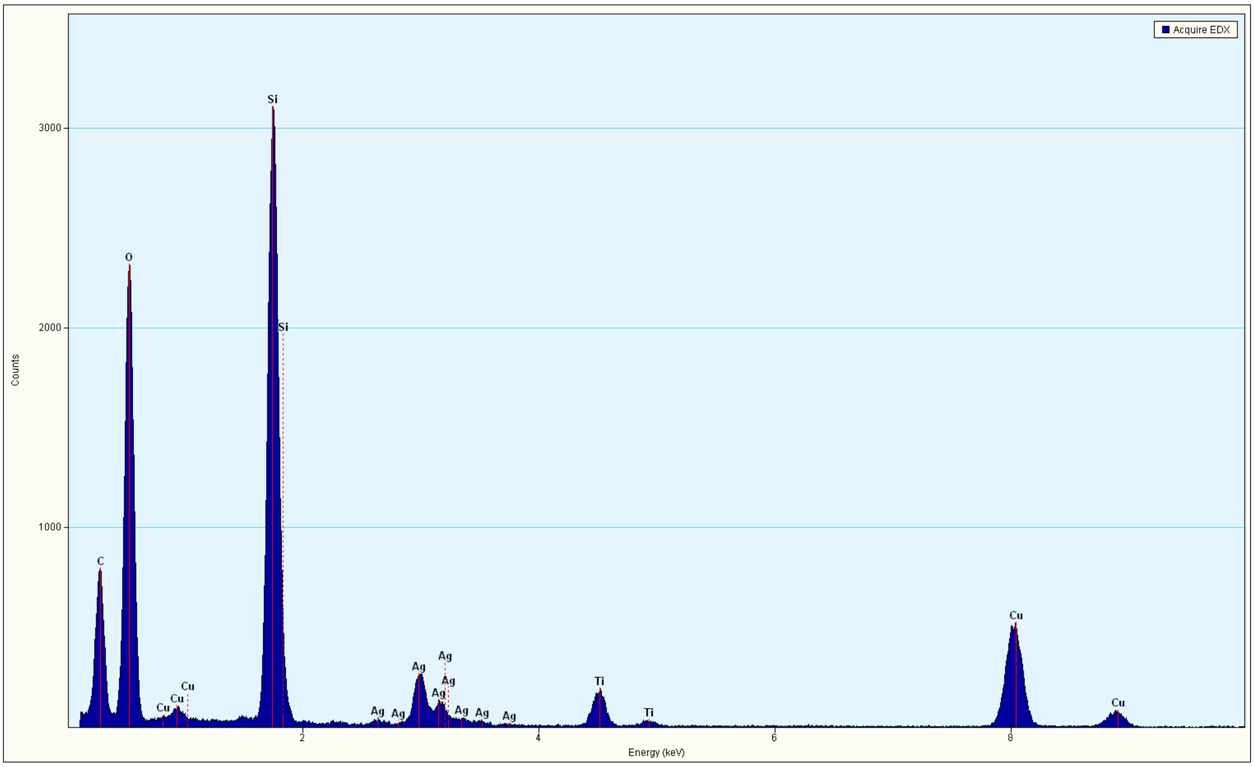
Acquire EDX
Si
O
Si
C
Cu
Cu
Cu
Ag
Ag
Ag
Ag
Ag
Ag
Ag
Ag
Ti
Ti
Cu
Cu
3000
2000
1000
Counts
2
4
6
8
Energy (keV)

b)

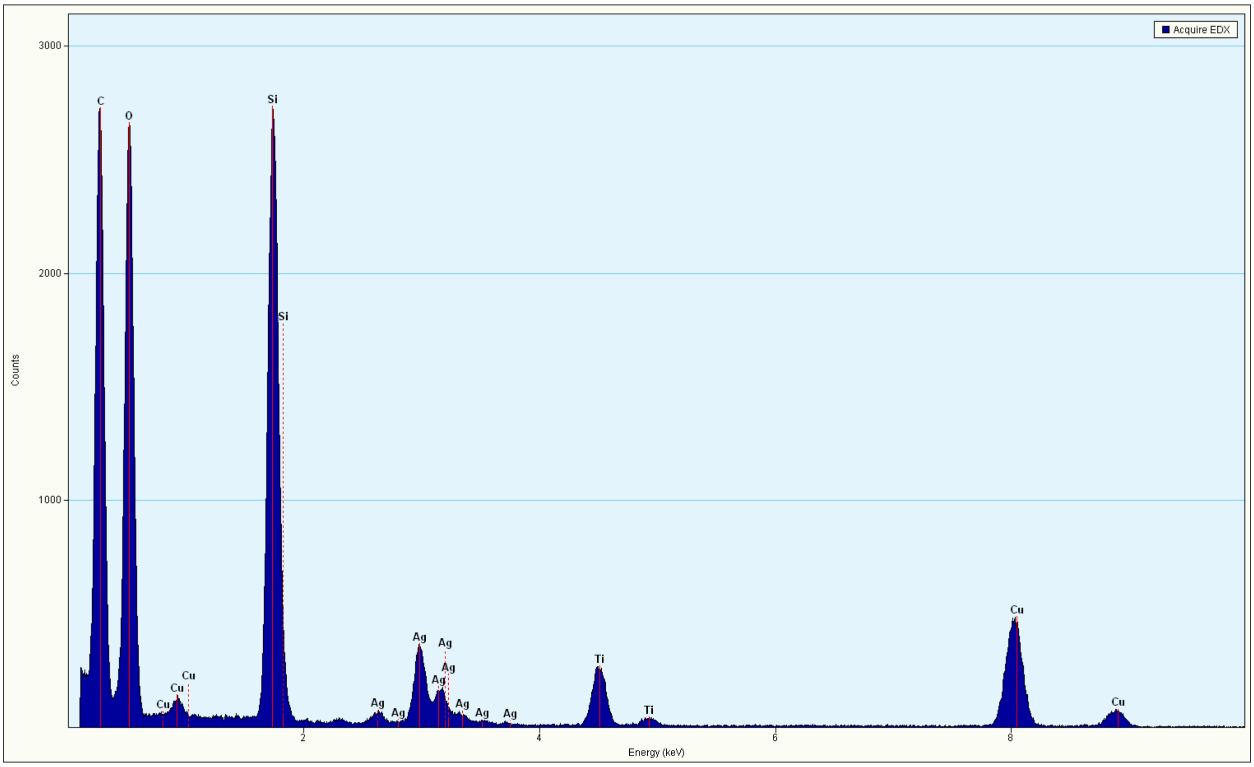
Acquire EDX
C
O
Si
Si
Cu
Cu
Cu
Ag
Ag
Ag
Ag
Ag
Ag
Ag
Ag
Ti
Ti
Cu
Cu
3000
2000
1000
Counts
2
4
6
8
Energy (keV)

c)

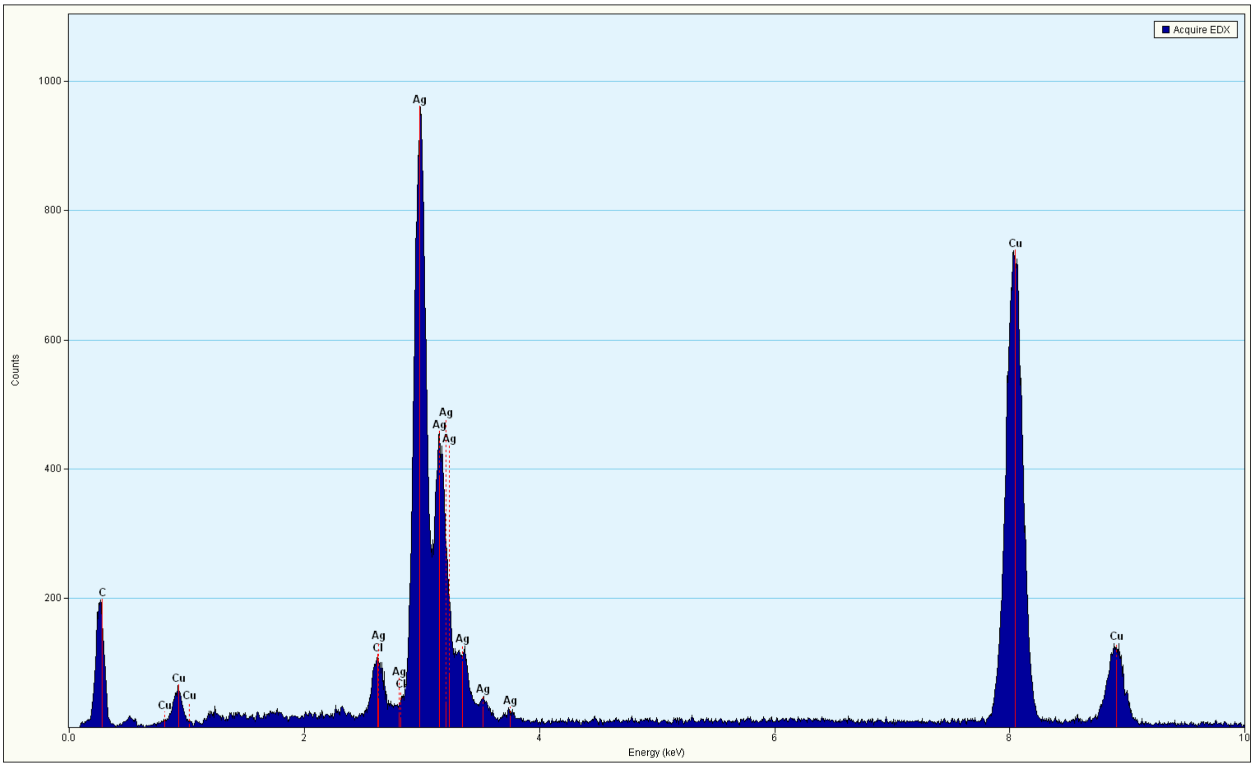

d)

Figure S4. EDX images of SAg (a); STrAg (b); STbAg (c) and AgNPs (d) samples

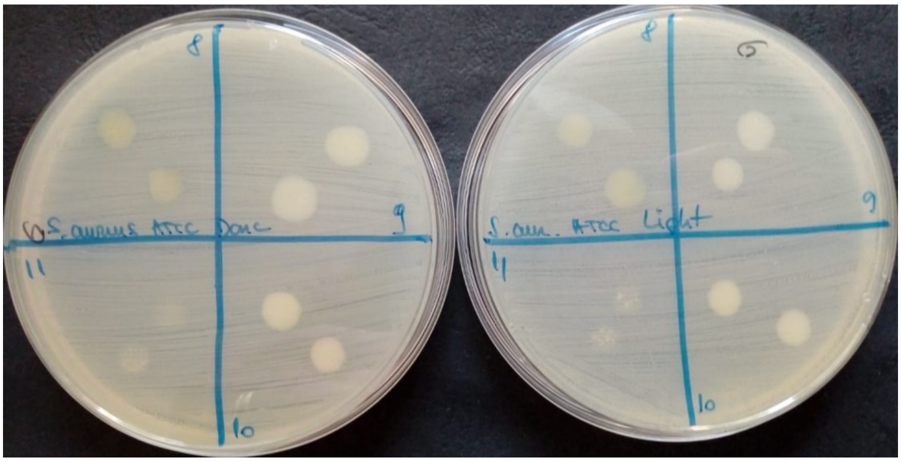

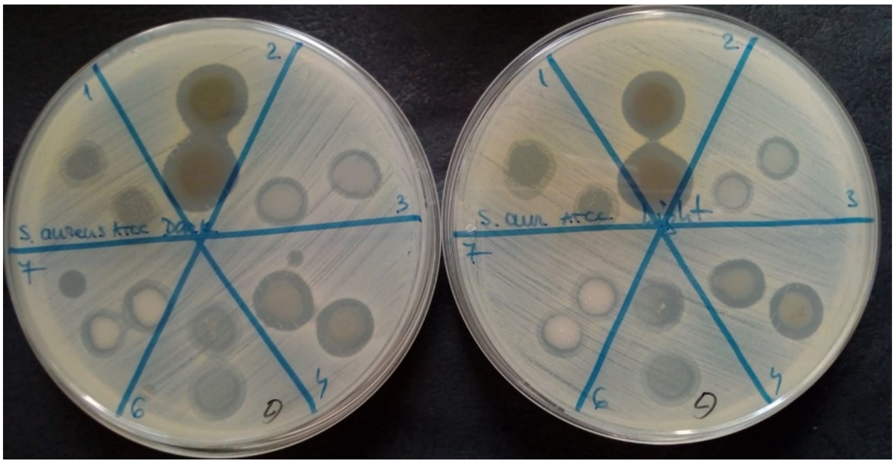

Figure S5. Growth inhibition zone diameters obtained after placing the sample spot on solid medium streaked with microbial inoculum of *Staphylococcus aureus* ATCC 25923;
(Left – darkness condition; right – light condition).

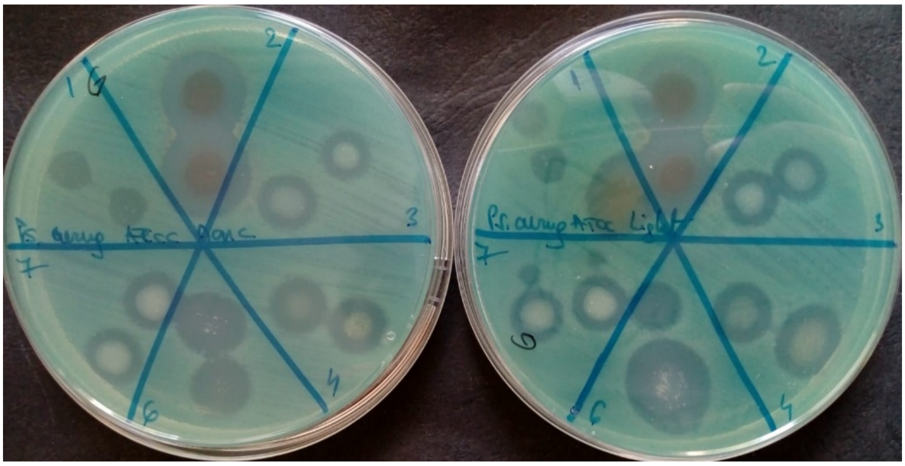

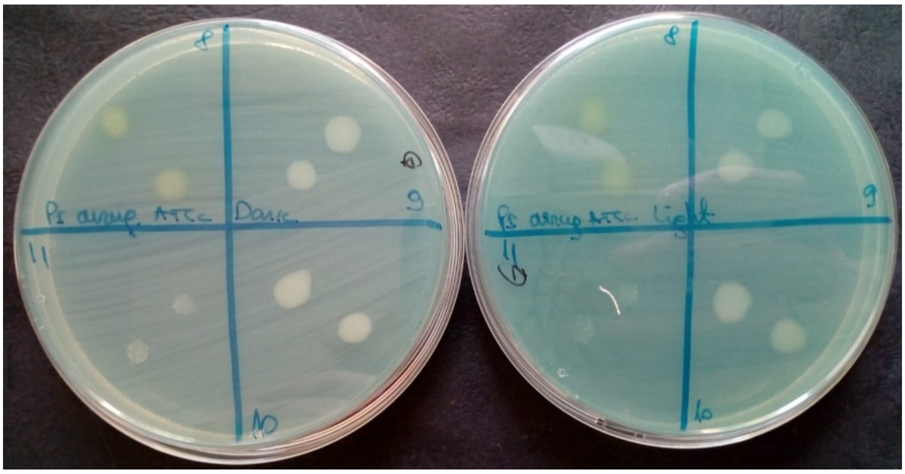

Figure S6. Growth inhibition zone diameters obtained after placing the sample spot on solid medium streaked with microbial inoculum of *Pseudomonas aeruginosa* ATCC 27853;

(Left – darkness condition; right – light condition).

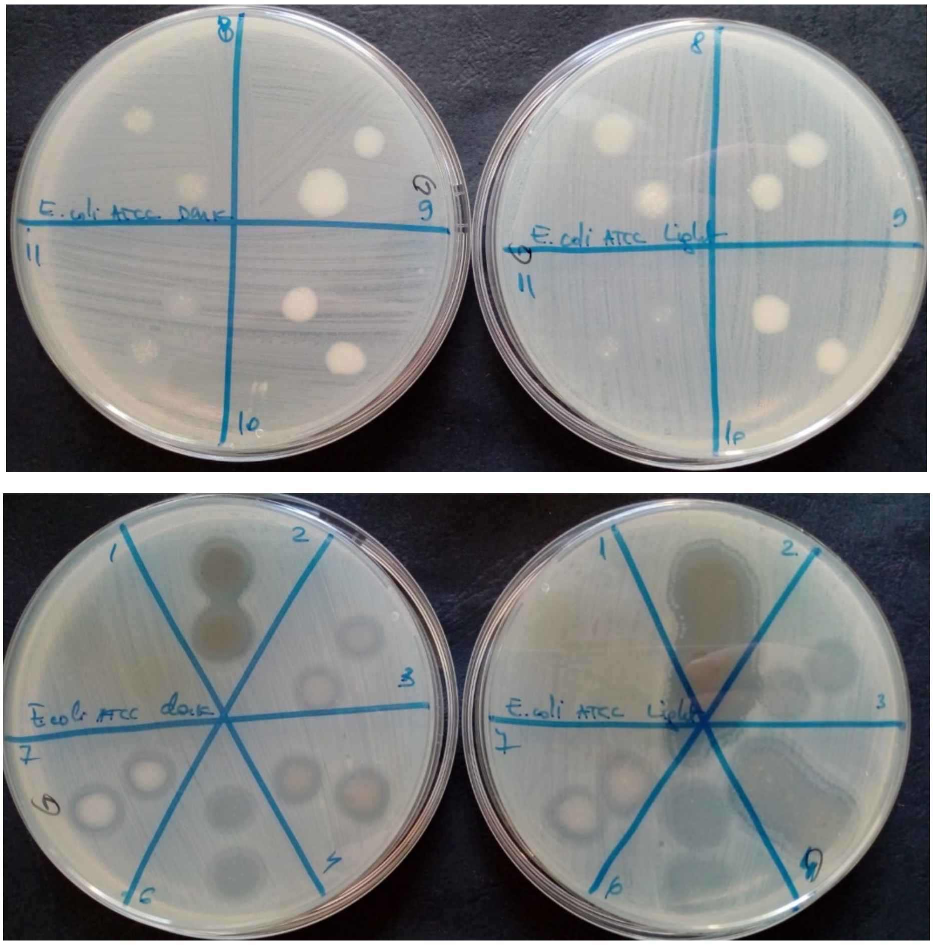

Figure S7. Growth inhibition zone diameters obtained after placing the sample spot on solid medium streaked with microbial inoculum of *Escherichia coli* ATCC 25922.

(Left – darkness condition; right – light condition).

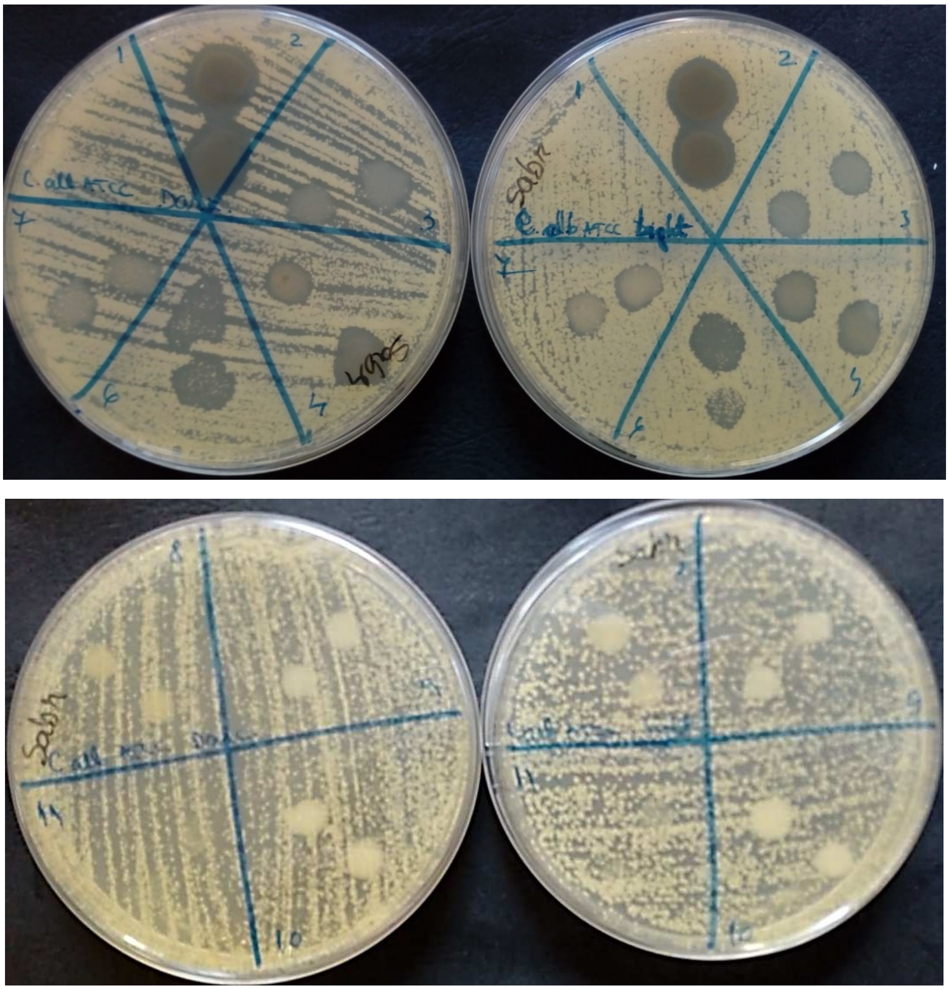

Figure S8. Growth inhibition zone diameters obtained after placing the sample spot on solid medium streaked with microbial inoculum of *Candida albicans* ATCC 10231;
(Left – darkness condition; right – light condition).

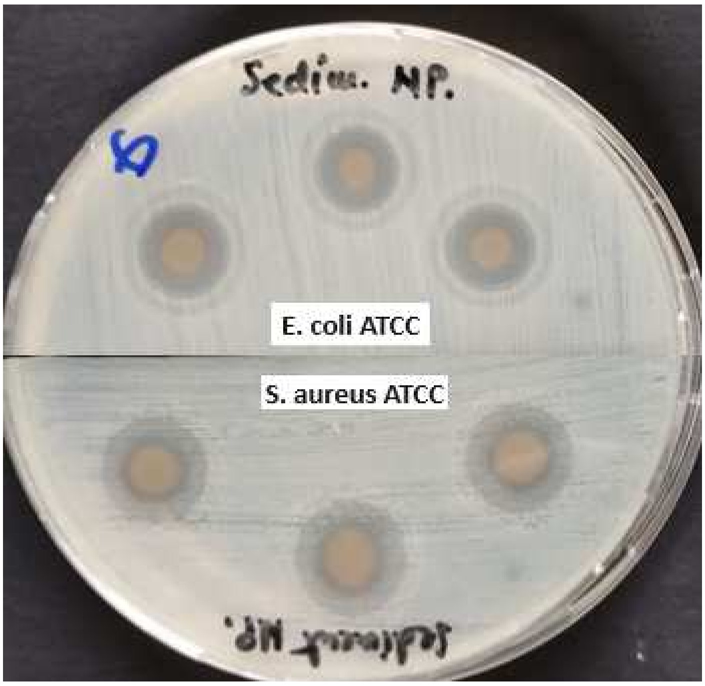

Figure S9. Growth inhibition zone diameters obtained in dark after placing theAgNPsi sample spot on solid medium streaked with microbial inoculum of *Escherichia coli* ATCC 25922 and *Staphylococcus aureus* ATCC 25923
